# Precision nutrition in weight loss and neuroendocrine control of people with obesity: The study protocol of a factorial randomised controlled trial (GenON Programme)

**DOI:** 10.1111/dom.70414

**Published:** 2026-01-02

**Authors:** Karla P. Balbino, Ana Claudia P. Kravchychyn, Flávia G. Cândido, Mariana de Moura e Dias, Tiago Antônio de O. Mendes, Leandro Licursi de Oliveira, Luiza Carla V. Castro, Josefina Bressan, Ana Laura de la Garza, Fermín I. Milagro, María A. Zulet, Helen Hermana M. Hermsdorff

**Affiliations:** ^1^ Laboratory of Clinical Analysis and Genomics, Department of Nutrition and Health Universidade Federal de Viçosa Viçosa Brazil; ^2^ Laboratory of Energy Metabolism and Body Composition, Department of Nutrition and Health Universidade Federal de Viçosa Viçosa Brazil; ^3^ Clinical Nutrition Didactic Laboratory, Department of Nutrition and Health Universidade Federal de Viçosa Viçosa Brazil; ^4^ Laboratory of Molecular Biotechnology, Department of Biochemistry and Molecular Biology Universidade Federal de Viçosa (UFV) Viçosa Brazil; ^5^ Laboratory of Immunochemistry and Glycobiology, Department of General Biology Universidade Federal de Viçosa (UFV) Viçosa Brazil; ^6^ Laboratory of Dietary Technique, Department of Nutrition and Health Universidade Federal de Viçosa Viçosa Brazil; ^7^ Institute for Obesity Research Tecnologico de Monterrey Monterrey Nuevo Leon Mexico; ^8^ Department of Nutrition, Food Sciences and Physiology, Centre for Nutrition Research, Faculty of Pharmacy and Nutrition University of Navarra Pamplona Spain; ^9^ Navarra Institute for Health Research (IdisNA) Pamplona Spain; ^10^ Centro de Investigación Biomédica en Red de la Fisiopatología de la Obesidad y Nutrición (CIBERobn) Instituto de Salud Carlos III Madrid Spain

**Keywords:** calorie restriction, epigenetics, microbiota, polymorphisms, satiety

## Abstract

**Aims:**

Precision nutrition, guided by genetic testing, has emerged as a promising approach for managing obesity. However, robust clinical trials testing its effectiveness in real‐world dietary interventions remain scarce. The GenOn Programme aims to evaluate whether tailoring nutritional care based on genetic risk for obesity enhances weight loss, satiety control, and metabolic outcomes in adults with overweight and obesity.

**Materials and Methods:**

The GenOn Programme is an 18‐week, 2 × 2 factorial, randomised controlled trial including 120 adults classified as high or low genetic risk for obesity (variants: FTO rs9939609 and rs1121980; MC4R rs1782313; LEP rs7799039). Participants are randomised to standard or satiety‐focused dietary counselling. Both groups receive five calorie‐restricted (−500 kcal/day), nutritionally balanced meal plans. The satiety arm additionally includes a high‐protein breakfast, daily granola supplementation and behavioural strategies. Assessments at baseline, Week 12, and Week 18 include weight loss, body composition, satiety perception, quality of life, cardiometabolic markers, (epi)genetics, inflammation, neuroendocrine regulation, and metagenomics.

**Results and Conclusions:**

The GenOn Programme is a randomised controlled trial to test a precision nutrition approach for overweight and obesity, integrating genetics, dietary strategies, and behavioural support. Findings may inform dietitians and healthcare systems on the clinical value of genetically guided nutritional care to improve outcomes in the treatment of overweight and obesity.

## 1 INTRODUCTION

Obesity is a chronic and progressive disease, defined by the excessive and abnormal accumulation of body fat,^1^ declared by the World Health Organization (WHO) as a global epidemic.^2^ Obesity is a multifactorial disease that is growing at alarming rates in Brazil and around the world, despite efforts to contain it. Information from the World Obesity Atlas^3^ indicates that, by 2030, 50% of adults worldwide will be living with body mass index (BMI) higher than 25.0 kg/m^2^, and 17% of men and 22% of women will be living with obesity.^3^ In Brazil, the same report also indicates that obesity affects 31% of the population, with projections indicating an increase of 33.4% among men and 46.2% among women by 2030.^3^

Obesity has a complex pathophysiological basis, characterised by dysfunction of hypertrophied mature adipocytes, imbalance in circulating concentrations of adipokines and non‐esterified fatty acids, oxidative stress and low‐grade chronic inflammatory processes.^4^, ^5^, ^6^ Furthermore, gut microbiota is considered an important factor influencing the development of obesity.^7^ Obese individuals often have microbiomes that increase energy extraction and fat accumulation.^8^ All these factors contribute to obesity‐related major complications, including type 2 diabetes mellitus (T2D), cardiovascular diseases, metabolic dysfunction‐associated steatotic liver disease (MASLD), respiratory disorders, nephropathy, musculoskeletal impairments, malignancies and psychological comorbidities.^9^

Although the obesity epidemic is associated with numerous changes in modifiable environmental factors,^10^, ^11^ the presence of genetic variants related to adiposity, food intake and metabolism can influence individual responses to the environment, as well as behaviour and food preferences.^11^ In this sense, clinical practice guidelines for the management of overweight and obesity highlight the importance of customised interventions and patient‐centred care.^12^ Precision nutrition represents an innovative approach that goes beyond conventional nutritional recommendations, providing personalised strategies based not only on phenotype but also on factors such as genetics, epigenetics, microbiome, biochemical markers, lifestyle and nutrition, which can help manage metabolic disorders such as obesity.^7^, ^13^

Unlike conventional nutritional practices, precision nutrition promotes better health outcomes by considering individual genetic variations that directly impact nutrient metabolism.^13^ In this sense, its strategy focuses on detecting individual biomarkers, allowing the application of more personalised and effective nutritional interventions to improve both prevention and treatment of obesity.^7^

The GenON Programme is a personalised nutritional intervention study conducted at the Universidade Federal de Viçosa in partnership with the Brazilian Ministry of Health, which has as its primary outcome to investigate the effect of a personalised diet that targets satiety control on body weight changes in carriers of single nucleotide polymorphisms (SNPs) in the FTO, MC4R and LEP genes. In this sense, since we believe that nutritional treatment of obesity may be more effective when the individual's genetic profile is considered, this randomised controlled trial aims to develop a genetically based personalised intervention for the nutritional care of people with overweight or obesity, representing an innovative approach to optimising the treatment of these individuals.

## 2 MATERIALS AND METHODS

### 2.1 Study design

The GenOn Programme is an 18‐week, 2 × 2 factorial, randomised controlled trial enrolling 120 adults of both sexes who present with overweight or obesity. The study of factors involves comparing genetic risk for obesity with nutritional interventions.

During screening visits, selected participants will be evaluated a priori for their genetic score and classified as low risk (LR) or high risk (HR) for obesity. Before intervention, participants will be randomly allocated into control or satiety intervention according to their risk classification, composing four experimental groups (control low‐risk group; control high‐risk group; intervention low‐risk group; intervention high‐risk group).

**FIGURE 1:**
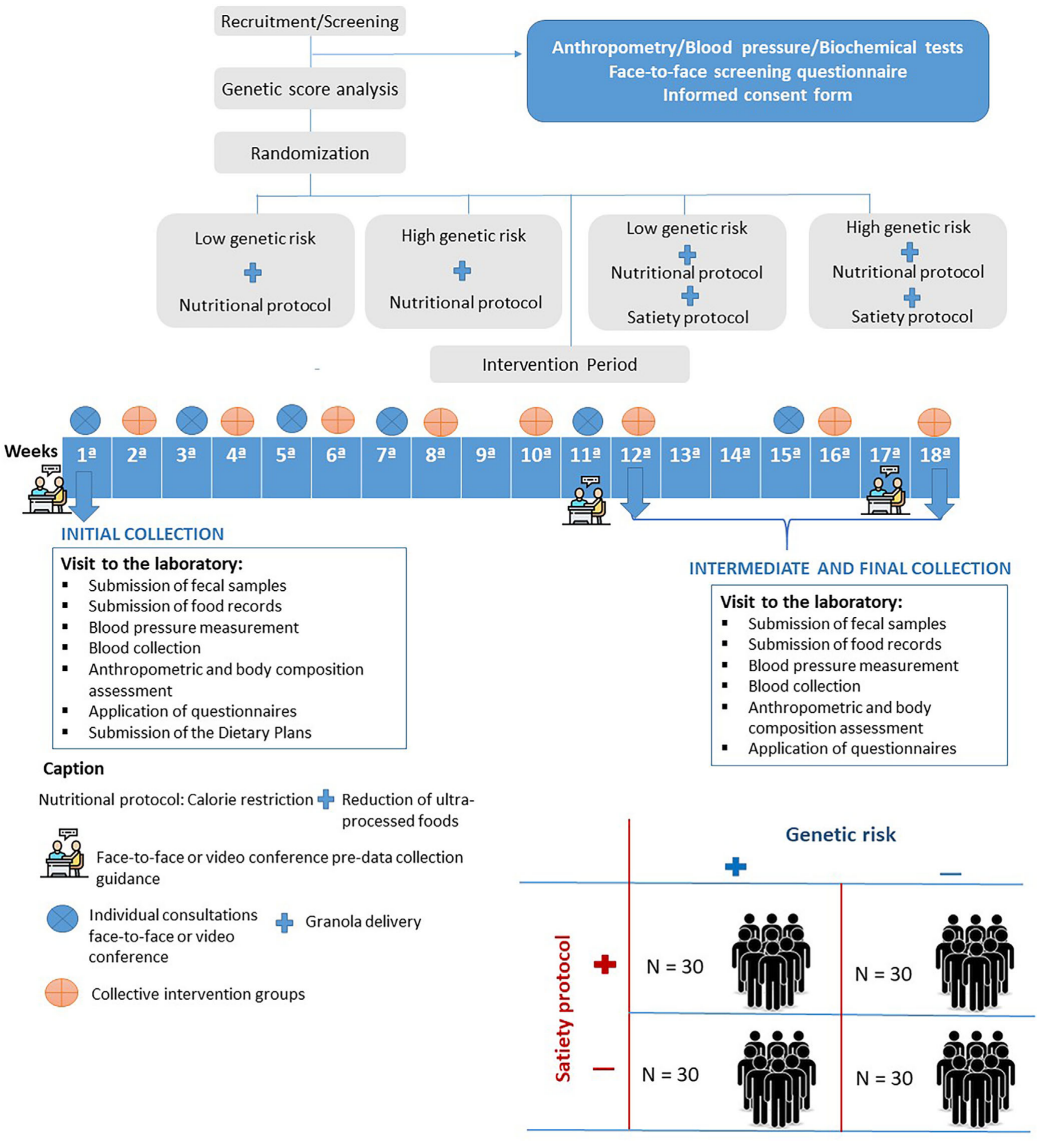
Study design of the GenOn Programme.

At the beginning and after 12 and 18 weeks, participants will attend the laboratory after a 10 to 12‐h fast and without water consumption, carrying 24‐h food records (three non‐consecutive days, including one weekend day) and faecal samples, to perform the study assessments. Participants will be evaluated for anthropometric and body composition analysis, questionnaire completion, blood sample collection and dietary prescription, in accordance with their assigned intervention group. Dietary interventions consist of 5 cal‐restricted nutritionally balanced plans (control intervention) or similar plans adapted to improve satiety, plus inclusion of granola (satiety intervention). All participants will receive biweekly individualised dietary counselling to promote healthier food behaviour, with emphasis on reducing the consumption of ultraprocessed foods, as recommended by Brazilian^14^, ^15^ and international^16^ guidelines. For the satiety group, granolas will be delivered, and compliance will be checked during individual counselling. Biweekly group psychological counselling, based on the experience of encounter groups, will be used to support self‐care, increase the sense of belonging to the programme and reduce dropout during the 18‐week follow‐up period. Furthermore, group counselling will be provided to address the psychological and behavioural aspects that influence the treatment of overweight and obesity, particularly among all treatment groups (Figure 1).

### 2.2 Ethics statement

This study was approved by the Human Research Ethics Committee of the Federal University of Viçosa (n° 7.283.411; approval date: 11 December 2024) and by the Brazilian Registry of Clinical Trials (ReBEC) (RBR‐8csbc2g). All individuals who agree to participate in this research will provide their written informed consent prior to the commencement of data collection.

### 2.3 Eligibility criteria

The study's inclusion criteria comprise: men and women aged 20–55 years who are overweight (BMI 27.0–29.9 kg/m^2^), have an elevated waist circumference (≥80 cm for women and ≥90 cm for men), and excess body fat (>30% for women and >20% for men), associated with at least one of the other components of the metabolic syndrome.^17^ Individuals with obesity (BMI ≥30 kg/m^2^) are also included if they present an elevated waist circumference and excess body fat, regardless of the other components.

The exclusion criteria are: women who are pregnant, breastfeeding, menopausal or with self‐declared intention to become pregnant during the trial; athletes; shift workers; clinical diagnosis of any mental, physical and/or intellectual disorder; food intolerances or allergies to the test products; type 1 diabetes mellitus; self‐report history of autoimmune diseases or use of immunosuppressant drugs; hormonal, digestive, inflammatory bowel, liver, kidney, pancreatic, cardiovascular or heart diseases; eating disorders; cancer; infectious episode within the past month; history of illicit drug use and/or alcohol intake >210 and >140 g of ethanol/week for men and women, respectively; planned or recent bariatric surgery; self‐reported weight fluctuations (5% of usual weight) in the past 3 months; habitual consumption of granola, nuts, whole grains or seeds (sesame, pumpkin and sunflower seeds, flaxseed) >30 g/day; adherence to vegan, vegetarian or dietary restriction diet (gluten and lactose); consumption of appetite and energy metabolism altering medications; use of vitamins, minerals and omega‐3 supplements above the Dietary Reference Intakes; use of fibre supplements (psyllium and inulin); adherence to a restrictive or therapeutic diet in the past 3 months; and current and anticipated enrolment in another research study.

### 2.4 Recruitment and screening

Participants will be recruited through advertising the study on radio stations and in newspapers, at health centres and healthcare plans, on posters and pamphlets and via institutional e‐mail and social media promotions, which will contain electronic links and contact information for the research team. When contacted by e‐mail or telephone, some questions will be asked to determine whether individuals meet the inclusion criteria. Then, they will be invited to a first visit at the Laboratory of Energy Metabolism and Body Composition of the Universidade Federal de Viçosa to confirm eligibility through the application of an in‐person screening questionnaire supplemented by information obtained in the previous screening, measurement of anthropometric measurements (weight, height and waist circumference) and blood pressure and biochemical tests (glucose, total cholesterol and fractions, triglycerides) from the last 3 months presented by the volunteer (when available) or solicited by the research team's. After confirming eligibility, the participant will be informed about the study procedures. If they agree, they must then sign the Informed Consent form. Following confirmation of participation in the study, a DNA sample will be collected for genotyping and determination of the genetic score.

### 2.5 Genetic score

To perform the genetic tests (SNPs panel—*FTO*: rs9939609 and rs1121980; *MC4R*: rs17782313; *LEP*: rs7799039), four previously well‐studied genetic variants associated with obesity and appetite regulation were selected. The swab technique will be used to collect cells from the oral mucosa. Participants will be instructed to rinse their mouths with 100 mL of distilled water previously, and the collection will be performed by scraping the inside of the cheeks with small sterile cytological brushes, making circular movements approximately 30 times. Then, the brushes will have the external portion of the stems cut and placed in 2 mL microtubes. The collected samples will be stored in an ultra‐low freezer at −80°C and analysed by real‐time polymerase chain reaction (PCR). The score will be assessed based on the presence or absence of the evaluated SNPs, and classification will be determined by the number of variant risk alleles at the Laboratory of Clinical Analysis and Genomics (UFV).

### 2.6 Outcomes

The primary clinical outcome will be estimated weight loss of 5%–10% of initial body weight, resulting in corresponding changes in BMI, waist circumference and body composition. Secondary outcomes will include improvements in indicators of quality of life, self‐esteem and quality of food intake, as well as serum cardiometabolic, oxy‐inflammatory, epigenetic, satiety and intestinal permeability markers.

### 2.7 Sample size calculation

For a sample calculation, analyses were performed assuming a weight loss of 5%–10% as the primary outcome, a significance level of 5%, a test power of 90% and an effect size of 0.50. Thus, considering a 30% sample loss, the sample size is 30 per group, totalling 120 participants. The G*Power 3.0.10 analysis programme was used, adopting an ‘a priori’ analysis using the variance test (ANOVA) for repeated measures.

### 2.8 Randomisation and allocation

Based on the genotypes, an unweighted risk genetic will be calculated, obtained by summing the number of identified risk alleles. Each genotype will be coded as 0, 1 or 2, according to the absence, presence in heterozygosity or homozygosity of the risk allele, resulting in a total genetic risk scoring (GRS) ranging from 0 to 8. From there, a sum of risk alleles will be made, and it will be pre‐categorised as high (≥3 variant risk alleles) or low (<3 variant risk alleles).^18^

Randomisation will be performed using the MinimPy® software (version 3.0), employing the minimisation method to ensure balance among the four groups in terms of sex, risk genetic (LR and HR), age range (20–39 years; 40–55 years) and BMI range (27.0–29.9 kg/m^2^; 30.0–34.9 kg/m^2^; 35.0–40.0 kg/m^2^).

### 2.9 Estimate of the energy requirements

The resting energy expenditure (REE) of all participants will be estimated using the Mifflin‐St. Jeor equation^19^ using the current body weight for the calculation. Then, the total energy expenditure will be determined by multiplying the REE by the individual's physical activity level. Caloric restriction will be defined by subtracting 500 kcal from each participant's estimated daily energy requirement.^14^, ^16^

### 2.10 Control group

The control group will receive a balanced diet with a macronutrient distribution and a caloric restriction of 500 kcal/day. Macronutrient distributions were determined according to guidelines for the treatment of overweight and obesity, which do not account for the individual's genotype.^14^, ^15^ The menus will be divided into six meals/day (breakfast, morning snack, lunch, afternoon snack, dinner and evening snack). They will be composed exclusively of foods with a lower degree of processing (whole foods, culinary ingredients and minimally processed foods).^20^ The menus will be calculated using an Excel spreadsheet based on the Nutritional Composition Tables of Foods Consumed in Brazil.^21^ Besides, participants will be instructed to drink at least 2 L of water/day and to avoid consuming oats, seeds and cashews throughout the study period.

### 2.11 Intervention group and satiety protocol

The intervention group will receive 500 kcal/day caloric restriction and a personalised diet designed to control satiety. The distribution of macronutrients was designed based on previous studies that investigated the effect of diets with different macronutrient distributions on body weight, metabolic markers and satiety sensation in individuals carrying genetic polymorphisms in the FTO, MC4R and LEP genes.^22^, ^23^, ^24^

The menus will follow exactly the same preparation and division criteria used for the control group. In addition to calorie‐restricted meal plans, an additional protocol focused on promoting satiety (satiety protocol) will be adopted, which consists of the following strategies: high‐protein breakfast (20% of the meal's calories coming from protein), daily consumption of a standardised granola (either a sweet or a salty version), meal frequency, mastication techniques and meal duration. The protocol as a whole was developed to act on both mechanical mechanisms and hormonal signalling pathways related to satiety. All strategies adopted were based on evidence from previous studies^25^, ^26^, ^27^ that demonstrated the effect of certain nutrients and foods on the modulation of satiety, including their influence on mechanical and hormonal processes and the subjective perception of satiety.

Participants will be instructed to consume the granola sachet daily at the most appropriate time for each one, which can be divided into two portions per day. The calories of the granola will be accounted within of the calculation of the caloric‐restricted diet. Granola will be used as a strategy to provide additional fibre and monounsaturated fats, as well as to stimulate mastication. Each serving of granola consist of 30 g of oats, 24 g of seeds, 15 g of cashew nuts and 5 g of cashew oil. Two flavours of granola will be developed: (1) sweet: with an additional of 5 g of brown sugar and 10 g of black raisins and (2) salty: with an additional of 0.15 g of salt and 0.3 g of sweet paprika.

The overview of the recommendations for the control and intervention groups is presented in Table 1.

**TABLE 1:** Overview of the GenOn Programme dietary intervention.

Outcomes	Control group	Satiety group
Calorie requirement	−500 kcal/day from energy requirement^20^	−500 kcal/day from energy requirement^20^
Dietary protein intake	15%–20% of energy^14^, ^15^	20%–25% of energy^14^, ^15^
Breakfast protein intake	10% of the daily protein intake^14^, ^15^	20% of the daily protein intake^24^, ^25^
Dietary carbohydrate intake	50%–60% of energy^14^, ^15^	45%–50% of energy^14^, ^15^
Dietary fibre intake	≤25 g/day^14^, ^15^	>30–35 g/day^14^, ^15^
Dietary fat intake	25%–30% of energy^14^, ^15^	25%–30% of energy^14^, ^15^
Dietary saturated fat intake	<10% of energy^14^, ^15^	<10% of energy^14^, ^15^
Dietary monounsaturated fat intake	<10% of energy^14^, ^15^	<10%–15% of energy^14^, ^15^
Dietary polyunsaturated fat intake	<8% of energy^14^, ^15^	<10% of energy^14^, ^15^
Intake of standardised granola (sweet or salty version)	Avoid consuming granola, oats, pumpkin, sunflower, flax and sesame seeds	Daily consumption of one sachet (30 g), which can be divided into two portions per day
Cashew nuts	Avoid consuming nuts	15 g/day
Individual consultations	Activities for the treatment of overweight and obesity	Activities for the treatment of overweight and obesity and techniques for the control of hunger and satiety

### 2.12 Collective intervention

In addition to the nutritional intervention, participants in both groups (control and intervention) will receive a continuous collective intervention consisting of eight meetings (the first six biweekly and the last two monthly), conducted by psychologists and nutritionists throughout the 18 weeks of the study. The collective intervention will follow Carl Rogers' person‐centred approach. From this perspective, facilitators will create a welcoming, judgement‐free environment that stimulates self‐awareness through reflection and careful clarification of doubts without offering direct advice. The principle is that, as participants broaden their understanding of themselves, viable solutions to their challenges emerge autonomously.^28^, ^29^

### 2.13 Monitoring and adherence to study protocol

Adherence to the protocol (in both the control and intervention groups) will be monitored through seven nutritional counselling consultations conducted biweekly during the first 2 months and subsequently monthly until the completion of the 18‐week study. The consultations will be conducted in person or via video conference, during which the consumption of granola, any possible adverse events, and the introduction or changes in usual medication doses for acute or continuous use will be recorded, and nutritional guidelines will be reinforced. In addition, during individual dietary consultations, adherence to granola consumption will be assessed by counting the returned empty packaging.

The control groups will also attend the monitoring visits and will undergo the same assessments as the intervention groups. During the study period, participants who do not adhere to the eating plan (in both the control and intervention groups), do not have good adherence to the satiety protocol (absence of consumption of the granola and intake of the high‐protein breakfast on at least five consecutive days), have a frequency of less than 75% in the activities of the collective intervention and nutritional consultations, or who experience any non‐transient adverse event and gastrointestinal symptoms that compromise health or quality of life related to the consumption of granola, will be discontinued from the study.

### 2.14 Data collection methods

Study outcomes will be assessed at three time points: the baseline, 12th week and 18th week after the study's start. Table 2 provides an overview of data collection and outcome measures.

**TABLE 2:** GenOn Programme data collection and outcome measures.

	Baseline	12th week	18th week
Data collection			
Sociodemographic, clinical and health questionnaire	X		
Faecal collection	X	X	X
Blood collection	X	X	X
Blood pressure measurement	X	X	X
Anthropometric (weight, height, waist and hip circumference, sagittal diameter) and body composition assessment	X	X	X
Three‐day food records	X	X	X
Habitual Physical Activity Questionnaire	X	X	X
Three‐Factor Eating Questionnaire‐R21 and Power Food Scale	X	X	X
Chrononutrion (Night Eating Questionnaire and Morningness‐Eveningness Questionnaire) and sleep quality (Pittsburgh Sleep Quality Index)	X	X	X
Gastrointestinal Symptom Rating Scale and Bristol Scale	X	X	X
36‐Item Short Form Survey	X	X	X
Outcome measures			
Weight loss; difference in BMI, waist circumference and body composition		X	X
Dietary intake, dietary pattern, NOVA classification and plant‐based diet indices	X	X	X
Eating behaviour and hedonic hunger	X	X	X
Blood chemistry analysis (blood count, lipid profile, fasting blood glucose, glycated haemoglobin, kidney and liver function and markers of oxy‐inflammation, neuroendocrine control of satiety and intestinal permeability)	X	X	X
Genomic DNA analysis	X	X	X
Gene expression analysis	X	X	X
Faecal short‐chain fatty acids and faecal potential of hydrogen	X	X	X
Metagenomic analysis	X	X	X
Gut health	X	X	X
Physical activity	X	X	X
Eating behaviour and hedonic hunger	X	X	X
Quality of life	X	X	X

Abbreviation: BMI, body mass index.

### 2.15 Blood pressure

Systolic and diastolic blood pressure will be measured using an automatic arm monitor (G‐Tech®, model BSP11, Accumed Medical‐Hospital Products Ltd., São Paulo, Brazil) with an accuracy of 3 mmHg. Blood pressure will be measured in both arms after a 5‐min rest period, and the measurement will be repeated in the arm showing the higher initial value.^30^ The result considered for analysis will be the mean of the last two readings and classified according to the cutoff points of the Brazilian Society of Hypertension.^31^

### 2.16 Anthropometric measurements and body composition

Body weight and body composition will be determined using a tetrapolar electrical bioimpedance equipment (InBody, model 230, BiospaceCo. Ltd), following the clinical protocol: 10–12 h of fasting for solid foods, 4 h for liquids, no alcohol or physical exercise in the 24 h prior to the test, no caffeine intake and with an empty bladder.

Height will be measured using a wall‐mounted stadiometer (Seca, model 206, Hamburg, Germany). The BMI will be calculated as weight in kilogrammes divided by the square of the height, and overweight/obesity was classified according to the cutoff points of the WHO.^32^

Waist, hip, neck, arm, thigh and calf circumferences will be measured with a flexible and inelastic measuring tape, with an accuracy of 0.1 cm, following standardised recommendations. Waist circumference will be measured midway between the iliac crest and the last rib. The sagittal abdominal diameter will be measured with an abdominal calliper (Holtain Kahn Abdominal Calliper®) with a mobile shaft and 0.1 cm subdivision. The measurement will be taken at the point of the largest abdominal diameter.^33^ All anthropometric measurements will be taken in triplicate, and the average of the two closest values will be used as the final estimated measurement.

### 2.17 Dietary intake

Data information from 24‐h food records completed into three sets of food records during the study (baseline, 12 and 18 weeks after the start of the study) will be entered into the Erica‐REC24h software.^34^ Proper recording of food and beverage intake, as well as estimating food portion sizes, will be explained to participants during the orientation session. The food diary must reflect all the food and beverages consumed by the participants within the specified periods, including the description of each food item (type, variety and brand name), amounts, the time of day the meal is taken and the cooking method (e.g., boiled, fried and broiled). The research team will verify and check the completeness and accuracy of the recorded intake. Total energy intake and nutrient consumption will be computed using the Nutritional Composition Tables of Foods Consumed in Brazil.^21^

The dietary pattern of individuals will be estimated using principal components factor analysis, which uses nutritional composition and food similarity to group foods.^35^, ^36^ Furthermore, the foods consumed by participants will be classified according to their degree of industrial processing, using the NOVA classification.^37^ Finally, food quality will be evaluated using three indices: the overall plant‐based diet index (PDI), the healthy plant‐based diet index (hPDI) and the unhealthy plant‐based diet index (uPDI).^38^, ^39^

### 2.18 Serum and plasma biomarkers

After a 10‐ to 12‐h fast, blood samples will be collected at the specified time points (baseline, 12 and 18 weeks after the start of the study). A trained nursing team will collect approximately 30 mL of blood for analysis of complete blood count, lipid profile, fasting blood glucose, glycated haemoglobin, kidney and liver function and markers of oxidative stress, inflammation, neuroendocrine control of satiety and intestinal permeability.

### 2.19 Genomic DNA and gene expression analysis

Genomic DNA will be extracted from peripheral blood cells using commercial DNA extraction kits. After obtaining the DNA, its concentration and purity will be determined spectrophotometrically. Total DNA methylation analysis will be performed using a commercial kit according to the manufacturer's recommendations.

Gene expression will be performed by extracting RNA from peripheral blood mononuclear cells ‐ PBMC using TRIzol® reagent (Invitrogen™) according to the manufacturer's recommendations. After RNA extraction, its concentration and purity will be determined spectrophotometrically. For retrotranscription, RNA aliquots will be standardised to 2 ng/μL. Ten microliters of sample will be added to 10 μL of master mix (2 μL of buffer solution, 0.8 μL of deoxynucleotide triphosphates (dNTP), 2 μL of random primers, 1 μL of reverse transcriptase, 1 μL of RNAse inhibitor and 3.2 μL of ultrapure water) and then incubated in a thermocycler at 25°C for 10 min, 37°C for 120 min, 85°C for 5 min, followed by cooling to 4°C. After double‐strand synthesis from total RNA, the complementary DNA (cDNA) concentrations of each sample will be determined spectrophotometrically and adjusted to the same concentration. For micro‐RNA (miRNA) analysis, 20 ng of total RNA will be reverse transcribed using the reverse transcription kit according to the manufacturer's protocol and the miRNA‐specific reverse transcription primers provided with the TaqMan MicroRNA Assay (Life Technologies). The miRNA‐specific cDNA will be amplified in triplicate with the PCR master mix and the respective manufacturer‐specific probe. The expression of genes and miRNA involved in cardiometabolic risk, inflammation, satiety and/or those modulated by nutritional intervention will be evaluated using a thermocycler. Gene expression levels will be assessed and normalised using *GAPDH* as an internal control following previously described protocols.^40^, ^41^ The fold change (2‐ddCt) in target genes, normalised to *GAPDH* and relative to the lowest expression profile, will be calculated for each sample according to the manufacturer's guidelines.

### 2.20 Faecal short‐chain fatty acids and faecal potential of hydrogen (pH)

Short‐chain fatty acids (SCFA) in faeces will be determined by high‐performance liquid chromatography (HPLC) after a cold extraction protocol.^42^ Faecal pH will be determined using a pH metre after dilution and homogenisation of faeces in ultrapure water.^43^

### 2.21 Metagenomic analysis

Total genomic DNA will be extracted from stool samples (300 mg) following a mechanical disruption (shaking and impact) and phenol/chloroform extraction protocol.^44^ Amplicons of the 16S rRNA V3‐V4 region will be generated using forward primer 341F (5′‐CCTAYGGGRBGCASCAG‐3′) and reverse primer 806R (5′‐GGACTACNNGGGTATCTAAT‐3′) and a barcoded primer set adapter for the Illumina NovaSeq platform (Illumina, San Diego, CA, USA).^44^ Samples will be loaded onto an Illumina flow cell for paired‐end sequencing reactions using the Illumina NovaSeq PE250 platform at Novogene Corporation on the University of California, Davis campus (Sacramento, CA, USA). Amplicons will be sequenced in a 2 × 250 bp NovaSeq run, using custom primers and sequencing procedures.^45^ Sequences obtained for all samples will be submitted to the Sequence Read Archive (SRA) database at the National Center for Biotechnology Information (NCBI) (http://www.ncbi.nlm.nih.gov/sra) under accession number PRJNA860372. Data processing and analysis will be performed using Mothur software v.1.44.3.^46^ Paired‐end sequences will be clustered into operational taxonomic units (OTUs). Each OTU represents a genetically unique group of biological organisms, and a sequence similarity cutoff point of 97% was adopted. The taxonomic classification of OTUs will be performed based on the SILVA v.138 database. The samples will be grouped according to the experimental groups and intervention times.

### 2.22 Eating behaviour and hedonic hunger

The Three‐Factor Eating Questionnaire‐R21 (TFEQ‐21)^47^ will evaluate the eating behaviour of the participants, and to assess hedonic hunger, the Power Food Scale^48^ will be used.

### 2.23 Gut health

The Gastrointestinal Symptom Rating Scale^49^ and the Bristol Scale^50^ will be used to evaluate participants' gut health.

### 2.24 Physical activity

The Habitual Physical Activity Questionnaire^51^ will be used to measure the level of physical activity of the participants.

### 2.25 Chrononutrion and sleep quality

The Night Eating Questionnaire^52^ and the Morningness‐Eveningness Questionnaire^53^ will be used to assess chrononutrition, and sleep quality will be assessed by the Pittsburgh Sleep Quality Index.^54^

### 2.26 Quality of life

The 36‐Item Short Form Survey (SF‐36)^55^ is an essential tool for assessing quality of life, with higher scores indicating better health.

### 2.27 Data handling and quality

The entire team involved in collecting data for this study will receive specific training and have prior experience in the field. The study's progress, protocol compliance, data validity and integrity and ethical considerations will be periodically assessed by a clinical trial monitor. Participants will be instructed to answer questions truthfully. An intention‐to‐treat (ITT) analysis will be used to include data from individuals who withdraw from the study for any reason. All participants will be followed for 18 weeks after treatment assignment.

After the questionnaires are administered, the responses will be checked for completeness and consistency. To ensure accurate data entry, two operators will enter the data independently, and the data entry system will feature pre‐programmed logic check functions to minimise transcription errors.

A unique numeric code will identify each participant. All data will be archived at the institution and accessible exclusively to the research team. Information from the questionnaires and consent forms will be kept in printed format, stored separately from other study data and identified by a specific numeric code.

### 2.28 Statistical analysis

Statistical analyses will be performed using SPSS software, version 24.0 (SPSS Inc., Chicago, IL, USA). Quantitative variables will be expressed as mean ± standard deviation (SD) or median (with 25th–75th percentiles), whereas qualitative variables will be presented as frequencies (percentages). Data normality will be assessed using the Kolmogorov–Smirnov test, and logarithmic transformation will be applied when distributions deviate from normality. Baseline differences between groups will be examined using the chi‐square test for qualitative variables and the independent‐samples Student's *t*‐test for quantitative variables.

A mixed ANOVA will be applied to determine the effects of groups (control low‐risk group; control high‐risk group, intervention low‐risk group, intervention high‐risk group), time (baseline, 12 weeks and 18 weeks) and genotype (LR and HR). Effect sizes will be calculated as partial eta‐squared (*ηp* ^2^), classified as small (0.01–0.05), moderate (0.06–0.13) or large (≥0.14).^56^ Bonferroni‐adjusted estimated marginal means will be used for post hoc comparisons. Variables violating homogeneity assumptions serão log10‐transformed. One‐way ANOVA will be used to compare changes in the evaluated variables between the control and intervention groups.

Linear and nonlinear regression models will be explored to estimate the association between variables with significant correlation. The statistical significance criterion will be *p* ≤ 0.05.

## 3 RESULTS AND DISCUSSION

Obesity is one of the main public health challenges today, recognised as a multifactorial condition with complex origins, involving interactions between genetic, metabolic, behavioural, environmental and social factors.^9^ Its prevalence has increased significantly in recent decades, reaching epidemic proportions in most countries, including Brazil.^57^

Obesity is associated with significant alterations in several clinical, biochemical and functional markers, reflecting its systemic and inflammatory impact.^58^ Excess adipose tissue, especially in the visceral region, contributes to a chronic state of low‐grade inflammation, characterised by increased proinflammatory cytokines and markers of oxy‐inflammation.^4^, ^5^, ^6^ This inflammatory state, together with the imbalance in gut microbiota also observed in obesity, leads to a deficiency in satiety signalling, resulting in increased intestinal permeability, resistance to leptin and insulin, ghrelin dysfunction and dysregulation of dopamine, serotonin and neuropeptides.^59^

Furthermore, changes in the lipid profile,^60^ insulin resistance,^61^ and hormones involved in satiety and energy expenditure, such as leptin, ghrelin and adiponectin,^62^ are observed. From a genetic perspective, variants associated with energy metabolism and appetite regulation have also been implicated in the predisposition to obesity.^11^ The integrated assessment of these parameters can contribute to a more comprehensive understanding of the mechanisms underlying obesity and to the development of more personalised therapeutic strategies.

Thus, precision nutrition represents an emerging approach that seeks to personalise nutritional recommendations based on individual genetic, metabolic and phenotypic characteristics, aiming to optimise health, prevent disease and enhance therapeutic response.^7^, ^13^ Unlike generalised dietary guidelines, this strategy integrates multiple components, including nutritional genomics, metabolomics, gut microbiota analysis, clinical and biochemical biomarker assessment, medical history, lifestyle factors and environmental and cultural factors.^63^, ^64^ Despite this potential, precision nutrition still requires more robust trials to assess its applicability, as well as its facilitators and challenges. For this reason, we believe that our study makes a significant contribution to filling part of this gap.

Finally, the application of GRS has gained relevance in precision nutrition, allowing the identification of individuals with greater genetic vulnerability and guiding specific preventive and therapeutic strategies. Furthermore, it contributes to the understanding of gene–environment interactions, highlighting how dietary and behavioural factors can modulate genetic risk.^65^ The GRS can be weighted or unweighted, differing in how each SNP contributes to the calculation of total risk. When known genetic variants are selected appropriately according to the study objectives, previous research has shown that both weighted and unweighted genetic risk scores are valuable tools for assessing genetic predisposition.^66^, ^67^, ^68^ Thus, other studies also adopt an unweighted approach, in which each risk allele is given for weight, regardless of whether the genotype is heterozygous or homozygous.^69^, ^70^

## 4 CONCLUSION

The GenOn Programme is a nutrition precision approach guided by genetic testing, which can achieve better efficacy in the treatment of overweight and obesity, demonstrating to the nutritionist and the multidisciplinary team the clinical value of genetically guided nutritional care in improving outcomes for this population.

## CONFLICT OF INTEREST STATEMENT

The authors declare no conflicts of interest.

## Data Availability

Data sharing not applicable to this article as no datasets were generated or analysed during the current study.
